# Academic benchmarks for leaders in Otolaryngology - Head & Neck Surgery: a Canadian perspective

**DOI:** 10.1186/s40463-020-00419-4

**Published:** 2020-05-06

**Authors:** Terence Fu, Vincent Wu, Paolo Campisi, Ian J. Witterick, Yvonne Chan

**Affiliations:** Department of Otolaryngology - Head & Neck Surgery, University of Toronto, Toronto, Canada

**Keywords:** Otolaryngology, Head & neck surgery, Leadership, H-index, Academic benchmark, Research productivity, Gender

## Abstract

**Background:**

The present study summarizes the demographics, subspecialty training, and academic productivity of contemporary leaders in Canadian Otolaryngology – Head & Neck Surgery (OHNS) training programs across Canada.

**Methods:**

Demographic data regarding chairpersons (CPs) and program directors (PDs) were obtained from publicly-available faculty listings and online sources, and included employment institution, residency training, fellowship training status, gender, and years of post-graduate experience. Research productivity was measured using the *h-index* and number of publications, obtained from Scopus. Characteristics of CPs and PDs were compared using statistical analysis.

**Results:**

Cross-sectional data was obtained from a total of 27 CPs and PDs from 13 accredited OHNS training programs across Canada active on July 1, 2019. All academic leaders completed at least 1 year of fellowship training. Head and neck oncology represented 77% of CPs and 59% of academic leaders overall, while pediatric otolaryngology represented 43% of PDs. Females represented 11% of academic leaders. There was a significant association between location of residency training and employment, with 56% (15/27) of physicians working where they had trained (*p* = 0.001, Fisher’s exact test; φ = 2.63, *p* = 0.001). On average, individuals with a graduate (Master’s) degree had a significantly higher H-index (17.7 vs 7.4, *p* = 0.001) and greater number of publications (106 vs. 52, *p* = 0.02). Compared to PDs, CPs had a significantly higher average *h-index* score (14.5 vs. 8.14, *p* = 0.04) and accrued more years of post-graduate experience (29.7 vs. 21.3 years, *p* = 0.008). There were no differences in the proportions of CPs and PDs with graduate degrees. There appeared to be a decline in research productivity beginning 3 years after academic appointment.

**Conclusions:**

This cross-sectional overview of academic leaders in Canadian OHNS programs demonstrates the following key findings: 1) all leaders completed fellowship training; 2) head and neck surgical oncology was the most common fellowship training subspecialty; 3) leaders were likely to be employed at the institution where they trained; 4) a Master’s degree may be associated with increased research productivity; 5) there is a potential risk of decreased productivity after appointment to a leadership position; and 6) women are underrepresented in academic leadership roles.

## Background

In 2015, Eloy and colleagues [1] published a cross-sectional overview of academic leaders in Otolaryngology – Head & Neck Surgery (OHNS). This study examined the profile of academic leaders including department chairpersons (CPs), vice CPs, and program directors (PDs) across 99 OHNS programs in the United States. Their findings centered on research and publication impact, additional training including fellowships and graduate degrees, and gender distribution across leadership positions. The study noted that CPs had more years of experience, higher publication impact, and increased research funding, with low representation of women in leadership positions, accounting for only 3% of CPs and 15% of PDs [1].

A similar overview has not been conducted in the Canadian setting. Examination of the characteristics and productivity of Canadian academic leaders will provide an understanding of the pathways for promotion in a Canadian context. It may also reveal possible issues with diverse representation within academic leadership. Therefore, the aim of the present study is to summarize the demographics, subspecialty training, and academic productivity of contemporary leaders in OHNS training programs across Canada. A secondary objective is to evaluate the impact of academic appointment on research productivity.

## Methods

Demographic data on departmental CPs and PDs from 13 accredited Canadian OHNS programs were obtained from publicly-available faculty listings after July 1, 2019. Current leaders were identified from the Canadian Society of Otolaryngology – Head & Neck Surgery (https://www.entcanada.org/cso/canadian-departments-otolaryngology/) and Royal College of Physicians and Surgeons of Canada (http://www.royalcollege.ca/rcsite/documents/arps/otolaryngology-e) websites and validated using departmental websites.

Data included employment institution, location of residency and/or fellowship training, nature of subspecialty training, gender, and years of experience following medical school graduation. Two individuals (one PD and one CP) with general OHNS subspecialty training reported on faculty listings were categorized according to their focus of clinical practice (pediatric otolaryngology and otology, respectively) after consensus agreement among all authors. Research impact and productivity were measured using the *h-index* and average annual number of publications obtained from Scopus. The *h-index* is a composite score that incorporates both the number of publications and number of citations of publications for an individual [1–6]. Individuals with multiple listings on Scopus were cross-referenced and non-duplicate publications were summed. In the event of multiple listings, the highest reported *h-index* was adopted. Data unavailable from public listings were supplemented with direct communication with individual physician leaders.

Statistical analysis was used to explore the strength of correlation between training and employment location using a Cramér’s V. Characteristics were compared between chairs and program directors using a t-test for continuous variables and a Chi-Square or Fisher’s exact test for categorical variables. To achieve the secondary objective of evaluating the impact of academic appointment on research productivity, the average number of publications over a standardized measure of time before and after academic appointment was plotted and compared using a t-test. Inclusion criteria for this analysis included appointment prior to January 2018 (allowing at least 1.5 years of post-appointment data) and *h-index* ≥ 1. Data was aggregated using Microsoft Excel 2018 (Microsoft Corp., Redmond, Washington), and statistical analyses were performed using SPSS version 24.0 (SPSS Inc., Chicago, Illinois). A *p*-value less than 0.05 was considered statistically significant.

## Results

Data was gathered from all 13 accredited OHNS training programs, with information available from 27 academic leaders, summarized in Table 1. There were 13 CPs and 14 PDs (one training program had 2 PDs).

**Table 1:** Summary and comparison of leadership benchmarks between departmental chairpersons and program directors

	CPs n (%)	PDs n (%)	Total N (%)	***P***-Value*
**Gender**				0.02
Male	13 (100)	11 (79)	24 (89)	
Female	0 (0)	3 (21)	3 (11)	
**Rank**				0.008
Professor	10 (77)	3 (21)	13 (48)	
Associate Professor	3 (23)	6 (43)	9 (33)	
Assistant Professor	0 (0)	5 (36)	5 (19)	
**Years of Experience, Mean (SE)**	29.7 ± 1.8	21.3 ± 2.2	25.3 ± 1.7	0.008
**Master’s Degree, No. (%)**	5 (38)	4 (29)	9 (33)	0.51
**Fellowship, All Subspecialties**^**a**^	13 (100)	14 (100)	27 (100)	< 0.01
Head and Neck	10 (77)	6 (43)	16 (59)	
Pediatric Otolaryngology	0 (0)	6 (43)	6 (22)	
Facial Plastic & Reconstructive Surgery	2 (15)	1 (7)	3 (11)	
Rhinology	1 (8)	1 (7)	2 (7)	
Otology	1 (8)	1 (7)	2 (7)	
Laryngology	2 (15)	0 (0)	2 (7)	
***H-index*, Mean** ± **SE (range)**	11.2 ± 1.6 (0–30)	14.5 ± 2.5 (0–30)	8.1 ± 1.6 (0–18)	0.04

*Abbreviations*: *CPs* chairpersons, *PDs* program directors

*Comparison of characteristics between CPs and PDs using a t-test for continuous variables and Chi-square or Fisher’s exact test for categorical variables

^a^Sum of percentages may exceed 100% as some individuals completed multiple fellowships. Two individuals (one PD and one CP) with general OHNS subspecialty training reported on faculty listings were categorized according to their focus of clinical practice (pediatric otolaryngology and otology, respectively) after consensus agreement among all authors

**Fig. 1:**
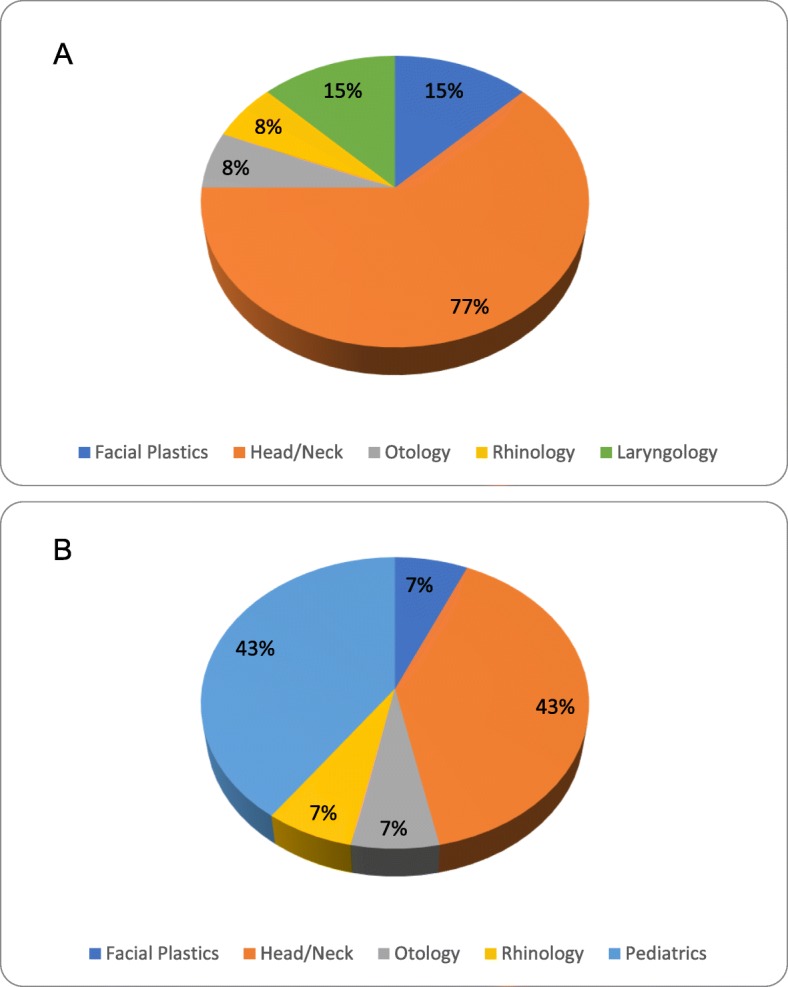
Fellowship subspecialty training among Canadian OHNS (**a**) chairpersons and (**b**) program directors

All academic leaders were fellowship trained. Among CPs, head and neck surgical oncology (77%), facial plastic surgery (15%), and laryngology (15%) were the most common areas of fellowship training (Fig. 1a). Among PDs, head and neck surgical oncology (43%) and pediatric otolaryngology (43%) were the most common fellowship-trained subspecialties (Fig. 1b). Head and neck surgical oncology (59%) was the most common area of subspecialty training among academic leaders overall.

**Fig. 2:**
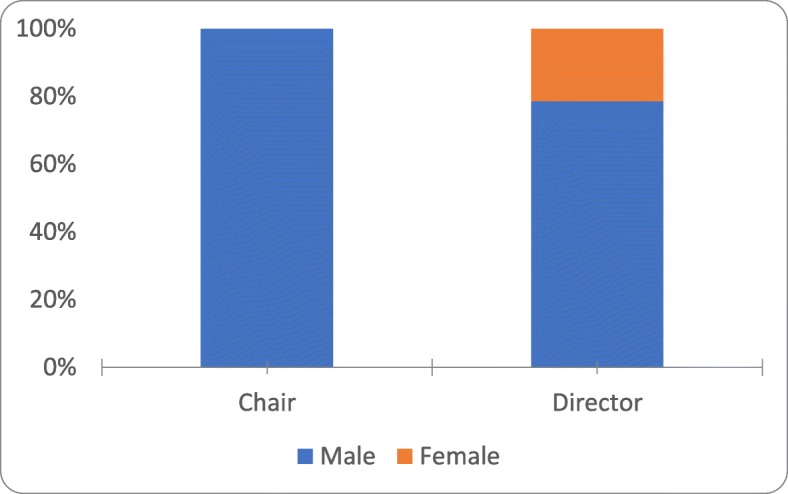
Representation of chairpersons and program directors by gender

Females represented 3 of 14 (21%) PDs, none of the CPs, and altogether only 11% of the academic leaders overall (Fig. 2). There was a significant association between location of residency training and employment, with 56% (15/27) of physicians working where they had trained (*p* = 0.001, Fisher’s exact test; φ = 2.63, *p* = 0.001).

**Fig. 3:**
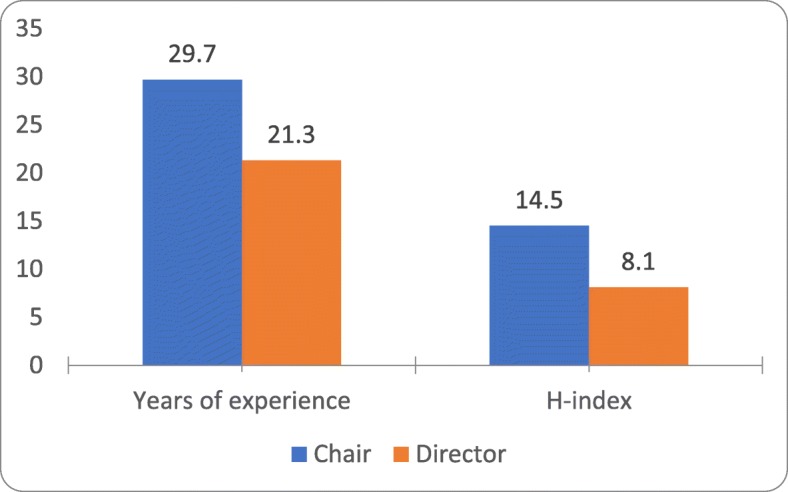
Comparison between years of post-graduate experience and *h-index* between chairpersons and program directors

**Fig. 4:**
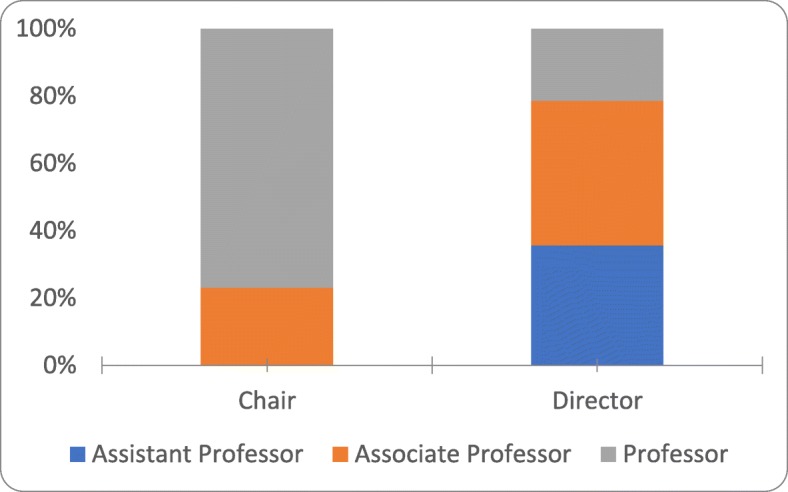
Comparison of academic ranks between chairpersons and program directors

Compared to PDs, CPs were more likely to have achieved the academic rank of full professor (77% vs. 21%). The remainder of the CPs were associate professors (23%). Overall, there was a significant difference in the composition of academic ranks (*p* = 0.008; Fig. 3). Not surprisingly, CPs accrued significantly more years of post-graduate experience (29.7 vs. 21.3 years, *p* = 0.008) and higher mean *h-index* scores (14.5 vs. 8.14, *p* = 0.04; Fig. 4).

In total, 9 academic leaders (33%) earned a graduate (Master’s) degree, including 5 CPs (38%) and 4 PDs (29%). There was no difference in the proportion of CPs and PDs with graduate degrees (*p* = 0.51). Individuals with a graduate degree had a significantly higher *h-index* (17.7 vs 7.4, *p* = 0.001) and average number of publications (106 vs. 52, *p* = 0.02) compared to those without a graduate degree.

**Fig. 5:**
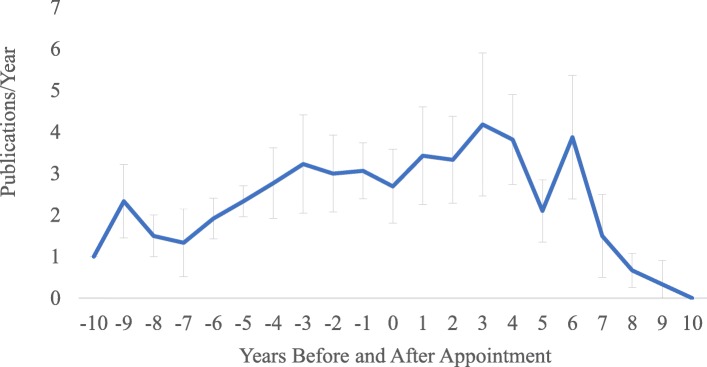
Impact of academic appointment on research productivity. Error bars represent standard errors of the mean. Time zero denotes time of academic appointment

The average number of publications over a standardized measure of time before/after academic appointment was plotted for 19 of 27 (70%) academic leaders meeting our inclusion criteria. We found that there were no significant differences before and after academic appointment (1.8 vs. 2.0, *p* = 0.64). However, we noted subjective trends of increasing research productivity leading up to appointment and peaking 3 years after appointment, followed by a notable decline in productivity (Fig. 5).

## Discussion

This is the first study to review the characteristics of academic leaders in Canadian OHNS programs. The primary goal of our study was to summarize the demographics, training characteristics, and academic productivity of contemporary leaders in OHNS training programs across Canada, with a secondary objective of evaluating the impact of academic appointment on research productivity.

Our study found that all CPs and PDs had at least 1 year of fellowship subspecialty training. This is not surprising as care is often subspecialized within academic centers in Canada. This finding may reflect the trend towards additional fellowship training as a prerequisite for academic practice over the past two decades [2, 7, 8]. The most common area of fellowship training was head and neck surgical oncology among both CPs and PDs. This finding was consistent with the study by Eloy et al., and reflects the historical influence and longevity of head and neck surgical oncology as the one of the earliest OHNS subspecialties to emerge [1, 2]. Pediatric otolaryngology was also heavily represented among PDs, and may reflect the fact that most academic pediatric otolaryngologists are remunerated through an alternate funding plan. In particular, salary-based compensation may afford pediatric subspecialists comparatively greater time and opportunity to pursue administrative and academic leadership roles. The impact of physician renumeration on academic involvement and research productivity was not formally evaluated in this study due to lack of publicly available data and could be explored with future research.

CPs had a significantly greater number of publications, a higher *h-index*, more years of post-graduate experience, and a higher proportion of full professors compared to PDs. Again, this finding was consistent with the previous study of U.S. OHNS programs [1]. This finding is not surprising as CPs typically represent more senior physicians with more clinical and academic experience. In several instances, previous PDs have transitioned to the role of CP over time.

Our study found that academic leaders with a Master’s degree had, on average, a significantly higher *h-index* score and greater number of publications, suggesting an added value to graduate-level training for acquiring additional skills and increasing academic productivity. However, the potential benefits of a Master’s degree must be balanced with the significant time and monetary investment, and therefore remains a highly individual- and institutional-dependent decision.

The secondary objective of this study was to examine the impact of academic appointment on research productivity, as measured by average annual publication rates before and after academic appointment. Although there was no significant difference in productivity between periods, we observed a trend of increasing productivity prior to appointment that peaked at 3 years post-appointment. In addition, there was a general decline over time beyond 3 years post-appointment. The observed trends must be interpreted cautiously due to the small sample size. One could speculate that the observed peak at 3 years post-appointment may correspond with an institutional three-year review or the completion of previous work leading up to the academic appointment. The subsequent decrease in productivity may be secondary to the increased administrative responsibility associated with leadership roles. On the other hand, some leaders were successful in maintaining their research productivity by incorporating new research interests in complementary areas such as medical education or curriculum development. The impact of administrative burden on productivity has particular importance to aspiring leaders considering the optimal time to pursue a leadership appointment.

Data published by the Canadian Medical Association (2018) has estimated that females represent 23% of practicing OHNS surgeons in Canada [9]. Despite females accounting for a minority of Canadian OHNS surgeons, our study found that even fewer still are represented among academic leaders and, more broadly, in academic otolaryngology. A review of faculty listings across Canadian academic centers, at the time of this writing, revealed that females represent approximately 19% of all academic otolaryngologists across Canada. Furthermore, our present study demonstrated that females represented only 11% of Canadian OHNS leaders overall. This figure is consistent with U. S studies which found that females were represented in 7 to 10% of American OHNS academic leadership roles [1, 10]. Choi et al. surveyed over 10,000 American Academy of Otolaryngology – Head & Neck Surgery members in 2010 and found that women represented only 4% of CPs and 12% of PDs [10]. In contrast to our study, Choi et al. noted that women were, in fact, proportionally represented after accounting for the number of females practicing OHNS. They also noted that women were proportionally represented in journal and special society leadership positions, and that female OHNS surgeons were relatively younger compared to their male counterparts, with over 63% of females under the age of 45 years [10]. This discrepancy can be explained by geographic variation in training, certification, employment opportunities, and patient population.

Despite the underrepresentation of females in otolaryngology and academic leadership, there is evidence that the gender proportions are becoming more balanced. The 23% female proportion of Canadian OHNS surgeons shown in 2018 CMA report represents a significant increase from the previous figure of less than 10% in the early 2000’s [9]. Representation is expected to further increase as females currently outnumber males entering medical school (56%), and represent approximately 40% of Canadian OHNS residents and 47% of surgical residents [11, 12]. We must continue to monitor the representation of females in our specialty and ensure equitable representation through ongoing efforts to attract and retain women in leadership positions.

### Study Limitations

The cross-sectional study design provided a snapshot of the current academic leadership profile and did not examine trends over time or describe the characteristics of other otolaryngologists across Canada. Data on *h-index* and academic productivity were gathered from Scopus similar to the methods of Eloy et al. [1]; these data are subject to potential variability depending on the type of citation tool used (i.e. web of science, Google scholar, etc.) [13]. Moreover, the study was not designed to consider confounding variables such as factors impacting choice of training, employment location, and interest in research. Furthermore, the study did not examine other aspects of leadership such as participation in teaching, committees, and clinical leadership roles. A more thorough review of leaders’ curriculum vitae would have captured a more robust assessment of their academic productivity. Future qualitative studies with a survey design and/or semi-structured interviews could also be used to capture more granular aspects of academic leadership.

## Conclusion

This study provides a cross-sectional overview of academic leaders in Canadian OHNS programs, and demonstrated the following key findings: 1) fellowship training was universal; 2) head and neck surgical oncology is the most common fellowship training subspecialty; 3) leaders were more likely to be employed at the institution where they trained; 4) a graduate (Master’s) degree may be associated with increased research productivity; 5) there is a potential risk of decreased academic productivity after appointment to a leadership position; and 6) women are currently underrepresented in academic leadership roles.

## Data Availability

The datasets used and/or analyzed during the current study are available from the corresponding author on reasonable request.

## Abbreviations

CPs: Chairpersons
PDs: Program director
OHNS: Otolaryngology – Head & Neck Surgery
